# Role of eosinophilia in patients with recurrent/metastatic head and neck squamous cell carcinoma treated with nivolumab: Prediction of immune‐related adverse events and favorable outcome

**DOI:** 10.1002/cam4.6648

**Published:** 2023-10-30

**Authors:** Yushi Ueki, Shusuke Ohshima, Jo Omata, Yusuke Yokoyama, Takeshi Takahashi, Ryusuke Shodo, Keisuke Yamazaki, Arata Horii

**Affiliations:** ^1^ Department of Otolaryngology Head and Neck Surgery Niigata University Graduate School of Medical and Dental Sciences Niigata Japan

**Keywords:** eosinophilia, immune‐related adverse events, nivolumab, squamous cell carcinoma

## Abstract

**Introduction:**

Immune‐related adverse events (irAEs) are prognostic factors for patients on nivolumab. However, predictors of irAEs have not yet been identified. We aimed to investigate the predictors of irAEs occurrence and nivolumab discontinuation due to irAEs.

**Methods:**

Sixty‐two patients with recurrent/metastatic head and neck squamous cell carcinoma received nivolumab therapy between June 2017 and December 2020. Treatment outcome was compared between the groups with or without irAEs. The irAE (+) group was further divided by nivolumab discontinuation. Progression‐free survival (PFS) and overall survival (OS) were compared between the groups. Predictors of irAE occurrence were analyzed.

**Results:**

Twenty‐one patients (33.9%) developed irAEs, and six (28.6%) discontinued nivolumab due to severe irAEs. The irAE (+) group had significantly longer PFS and OS than the irAE (−) group (median PFS, 12.7 vs. 1.9 months; median OS, 33.1 vs. 12.8 months). The treatment outcomes in the discontinuation group were comparable to those in the non‐discontinuation group. The maximum absolute eosinophil count (AEC) during nivolumab therapy was significantly higher in the irAE (+) group than in the irAE (−) group (548.8 vs. 182) and higher in the discontinuation group than in the non‐discontinuation group (729.3 vs. 368.6). The receiver operating characteristic curve showed that the maximum AEC had a moderate‐to‐high accuracy for predicting irAE occurrence (area under the curve [AUC], 0.757) and nivolumab discontinuation (AUC, 0.893).

**Discussion:**

Monitoring AEC during nivolumab therapy may be useful in predicting irAE occurrence, nivolumab discontinuation, and disease prognosis.

## 1 INTRODUCTION

Immune checkpoint inhibitors (ICIs), including monoclonal antibodies against programmed cell death, have a significant effect on the treatment of recurrent/metastatic head and neck squamous cell carcinoma (R/M HNSCC).^1^, ^2^ As a remarkable feature, ICIs induce characteristic adverse events known as “immune‐related adverse events (irAEs)” that differ from those induced by traditional cytotoxic agents.^3^ irAE occurrence is associated with favorable treatment outcomes in various cancers.^4^, ^5^, ^6^ Furthermore, long‐term survival owing to durable response is achieved even in patients who discontinue ICI treatment because of irAEs.^7^, ^8^, ^9^ Severe irAEs may result in deterioration of QOL or treatment‐related death. Predicting the occurrence of irAEs and providing early interventions is important. However, it is not clear which patient populations are susceptible to irAEs and ICI discontinuation. On analyzing clinical data of patients with R/M HNSCC who received ICI treatment with nivolumab, we found that patients with a remarkable increase in blood eosinophil counts during ICI treatment and severe irAEs, such as colitis, interstitial pneumonia, and liver dysfunction, experienced nivolumab discontinuation. Eosinophil infiltration into tumor tissues and eosinophilia are favorable prognostic factors for various types of cancer.^10^, ^11^, ^12^ Regarding the relationship between eosinophil count and the risk of irAEs, several reports demonstrated that high baseline absolute eosinophil count (AEC) is a potential predictor of irAEs.^13^, ^14^ However, it has not been elucidated whether a chronological change in AEC during ICI treatment predicts the occurrence and severity of irAEs. Hence, this study aimed to evaluate the relationship between irAEs and the prognosis of HNSCC patients and to assess the predictive role of eosinophilia in irAE occurrence and nivolumab discontinuation due to irAEs.

## 2 METHODS

### 2.1 Patients and clinical data

This study was approved by the Institutional Review Board of Niigata University Hospital (No. 2019–0172). This study was conducted in accordance with the principles of the Declaration of Helsinki. We retrospectively analyzed the clinical data of patients with R/M HNSCC treated with nivolumab between June 2017 and December 2020.

We reviewed the patients' electronic clinical records and extracted data on age, sex, primary site, recurrent/metastatic site, Eastern Cooperative Oncology Group performance status (PS) score, and treatment outcomes. Programmed death‐ligand 1 (PD‐L1) expression in either surgical or biopsy specimens was evaluated by immunohistochemical testing (Dako 22C3 pharmDx, Agilent Technologies/Dako) and grouped according to expression levels, comprising levels of <1% and ≥1% in at least 100 evaluated tumor cells. Data on AEC were obtained from blood samples routinely collected after every nivolumab administration.

### 2.2 Assessment of treatment outcomes and immune‐related adverse events (irAEs)

Progression‐free survival (PFS) was defined as the time from the date of the first nivolumab administration to the date of disease progression or death. Overall survival (OS) was defined as the time from the start of nivolumab treatment to the date of death from any cause. IrAEs were evaluated according to the Common Terminology Criteria for Adverse Events, version 4.0. We divided the patients into two groups based on irAE occurrence: irAE (+) and irAE (−) groups. Furthermore, we divided the irAE (+) group into two groups based on nivolumab discontinuation owing to irAEs: discontinuation and non‐discontinuation groups. We compared the treatment outcomes between these groups.

### 2.3 Analysis of predictive factors for irAEs

To identify the potential predictive factors associated with irAE occurrence, we compared the following data between the irAE (+) and irAE (−) groups: baseline characteristics, maximum AEC during nivolumab therapy, and the time lag between the maximum AEC and irAE onset (irAE onset – maximum AEC). The negative and positive values of the time lag indicated that the maximum AEC followed and preceded the irAEs, respectively. We further evaluated the predictive capability of the maximum AEC for irAEs and nivolumab discontinuation using receiver operating characteristic (ROC) curves.

### 2.4 Statistical analyses

The cutoff date for the analyses of PFS and OS was August 31, 2021. Survival time was estimated using the Kaplan–Meier method and compared using log‐rank tests. Analysis of predictive factors was performed using Fisher's exact test or the Mann–Whitney U test. Statistical significance was set at *p* < 0.05. All statistical analyses were performed using EZR (Saitama Medical Center, Jichi Medical University, Saitama, Japan), a graphical user interface for R (The R Foundation for Statistical Computing, Vienna, Austria). Precisely, it is a modified version of the R commander designed to add statistical functions frequently used in biostatistics.^15^

## 3 RESULTS

### 3.1 Patient characteristics

Table 1 summarizes the baseline characteristics of the patients. Sixty‐two patients had a median age of 66 (range, 43–79) years. Fifty‐four (87.1%) patients were male; 33 had locoregional recurrence, whereas 29 had distant metastases alone. Regarding prior therapy, 42 (67.7%) patients received radiotherapy before nivolumab, whereas the remaining 20 (32.3%) did not. Regarding the chemotherapy lines administered before nivolumab, one line was administered to 36 (58.1%) patients, two lines, to 22 (35.5%) patients, and three or more lines, to 4 (6.5%) patients. The median duration of nivolumab administration was 3.0 (range, 0.5–50.3) months.

**TABLE 1:** Baseline characteristics.

Age, median (range)	66 (43–79)
Sex	
Male	54
Female	8
Primary site	
Oral cavity	14
Sinonasal sinus	13
Nasopharynx	4
Mesopharynx	8
Hypopharynx	13
Larynx	3
Others	7
Recurrent/metastatic site	
Locoregional recurrence	33
Distant metastasis alone	29
Performance status	
0	17
1	37
2	8
PD‐L1 expression level	
<1% or not available	46
1% ≤	16
Prior radiotherapy	
Yes	42
No	20
Number of chemotherapy lines before Nivolumab	
1	36
2	22
3 ≤	4
Duration of nivolumab administration, median (months, range)	3.0 (0.5–50.3)

Abbreviations: AEC, absolute eosinophil count; ALC, absolute lymphocyte count; ANC, absolute neutrophil count; CRP, C‐related protein; WBC, white blood cell.

### 3.2 irAE distribution

irAE distribution is summarized in Table 2. Twenty‐one of 62 (33.9%) patients had irAEs, and 26 irAEs showed overlaps. The median time from the start of nivolumab to irAE onset was 2.1 (range, 0.6–14.7) months (data not shown). Regarding irAE grade, 11 (42.3%) each had grade 1 and 2 irAEs, respectively, and four (15.4%) patients had grade 3 irAEs. Six of 21 (28.6%) patients discontinued nivolumab owing to severe irAEs (indicated by double asterisks): Three patients had grade 2 interstitial pneumonia, and one each had grade 3 colitis, increased liver enzyme levels, and oral mucositis. No grade 4 or 5 irAEs were observed.

**TABLE 2:** irAE distribution.

		irAEs			
		Total^a^	Grade 1	Grade 2	Grade 3
Any		26	11	11	4
Endocrine	Hypothyroidism	7	2	5	
	Hypophysitis	2	1		1
Skin	Rash	7	6	1	
Gastrointestinal	Colitis	2	1		1^b^
Hepatobiliary	Liver enzyme increased	1			1^b^
Pulmonary	Interstitial pneumonia	3		3^‡^	
Others	Oral mucositis	3	1	1	1^b^
	Arthritis	1		1	

Abbreviations: irAEs, immune‐related adverse events.

^a^ Data with overlaps.

^b^ irAEs caused discontinuation of nivolumab.

### 3.3 Treatment outcome according to irAE occurrence

**FIGURE 1:**
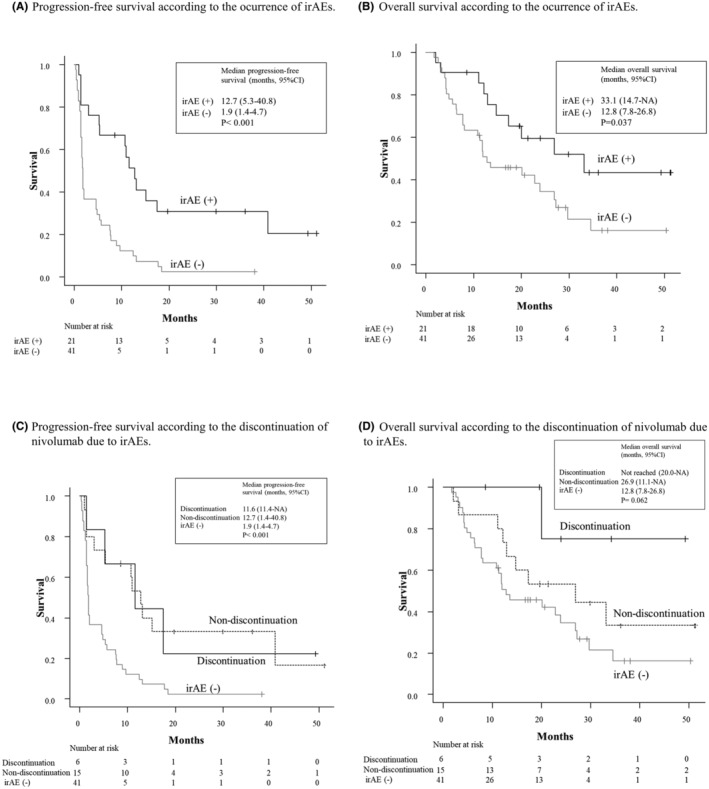
Survival analysis according to immune‐related adverse event (irAE) occurrence and nivolumab discontinuation owing to (irAEs). (A) Progression‐free survival (PFS) according to irAE occurrence: The median PFS period of patients with irAEs was significantly longer than that of patients without irAEs (12.7 vs. 1.9 months, *p* < 0.001). (B) Overall survival (OS) according to irAE occurrence: The median OS period of patients with irAEs was significantly longer than that of patients without irAEs (33.1 vs. 12.8 months, *p* = 0.037). (C) PFS according to nivolumab discontinuation: The median PFS patients who discontinued nivolumab owing to irAEs was comparable to that of patients who did not. Both were significantly longer in those with irAEs than those without irAEs (11.6 vs. 12.7 vs. 1.9 months, *p* < 0.001). (D) OS according to nivolumab discontinuation: The median OS period of patients who discontinued nivolumab was remarkably longer than that of the rest, although not at a significant level (not reached vs. 26.9 vs. 12.8 months, *p* = 0.062).

Figure 1 shows the survival rates according to irAE occurrence (A, B) and nivolumab discontinuation owing to irAEs (C, D). The median follow‐up time of the patients was 16.9 (range, 2.1–51.3) months. The median PFS and OS of all the patients were 3.8 (95% confidence interval [CI], 1.7–7.6) and 20.1 (95% CI, 12–27.2) months, respectively (data not shown). The irAE (+) group had significantly longer PFS (Figure 1A) and OS (Figure 1B) than the irAE (−) group (median PFS, 12.7 vs. 1.9 months, *p* < 0.001; median OS, 33.1 vs. 12.8 months, *p* = 0.037). The median PFS (Figure 1C) and OS (Figure 1D) in the discontinuation group were 11.6 months (95% CI, 11.4–NA) and not reached (95% CI, 20.0–NA), respectively, whereas those in the non‐discontinuation group were 12.7 months (95% CI, 1.4–40.8) and 26.9 months (95% CI, 11.1–NA), respectively. These results demonstrate that the discontinuation and non‐discontinuation groups had comparable treatment outcomes.

### 3.4 Details of patients who discontinued nivolumab owing to irAEs

Table 3 shows the details of six patients who discontinued nivolumab owing to irAEs. All patients were male. The irAEs causing nivolumab discontinuation were interstitial pneumonia (grade 2) in three patients and colitis (grade 3), elevated liver enzyme levels (grade 3), and oral mucositis (grade 3) in one patient each. Systemic steroid was administered to one patient with grade 3 colitis, whereas the other three patients with interstitial pneumonia received medications, such as antitussive drug, expectorant, and topical steroid, and the remaining two patients were carefully observed without interventions. All patients recovered from the irAEs. The AEC began to increase from the start of nivolumab treatment (data not shown), and the median maximum AEC was 730 (range, 541–1218). Three out of the six patients in the discontinuation group received sequential chemotherapy, whereas the other three did not. Nonetheless, two of the latter three patients survived for over 1 year.

**TABLE 3:** Details of patients who discontinued nivolumab owing to irAEs.

Case	Age	Sex	Primary site	irAE, grade	Treatment to irAEs	Baseline AEC	Maximum AEC	Sequential chemotherapy	Status (months from the start of nivolumab
1	61	Male	Oral cavity	**Colitis, 3**	Systemic steroid	193	580	None	AWD (43.7)
2	75	Male	Oral cavity	**Interstitial pneumonia, 2**	Medication	129	748	None	DOD (8)
3	66	Male	Maxillary sinus	**Interstitial pneumonia, 2**	Medication	99	868	S‐1	AWD (32.8)
4	57	Male	Hypopharynx	**Elevated liver enzyme level, 3**	Observation	188	1218	None	AWD (17.9)
5	68	Male	Hypopharynx	**Interstitial pneumonia, 2; rash, 1; colitis, 1**	Observation	222	711	S‐1, PTX	AWD (11.8)
6	64	Male	Oral cavity	**Oral mucositis, 3;** rash, 2; **hypothyroidism, 2**	Medication	31	541	S‐1	AWD (3.5)
						Median: 159	Median: 730		

*Note*: Bold: irAEs that led to nivolumab discontinuation.

Abbreviations: AEC, absolute eosinophil count; AWD, alive with disease; DOD, dead on disease; irAEs, immune‐related adverse events.

### 3.5 Predictive factors for irAEs and nivolumab discontinuation

**FIGURE 2:**
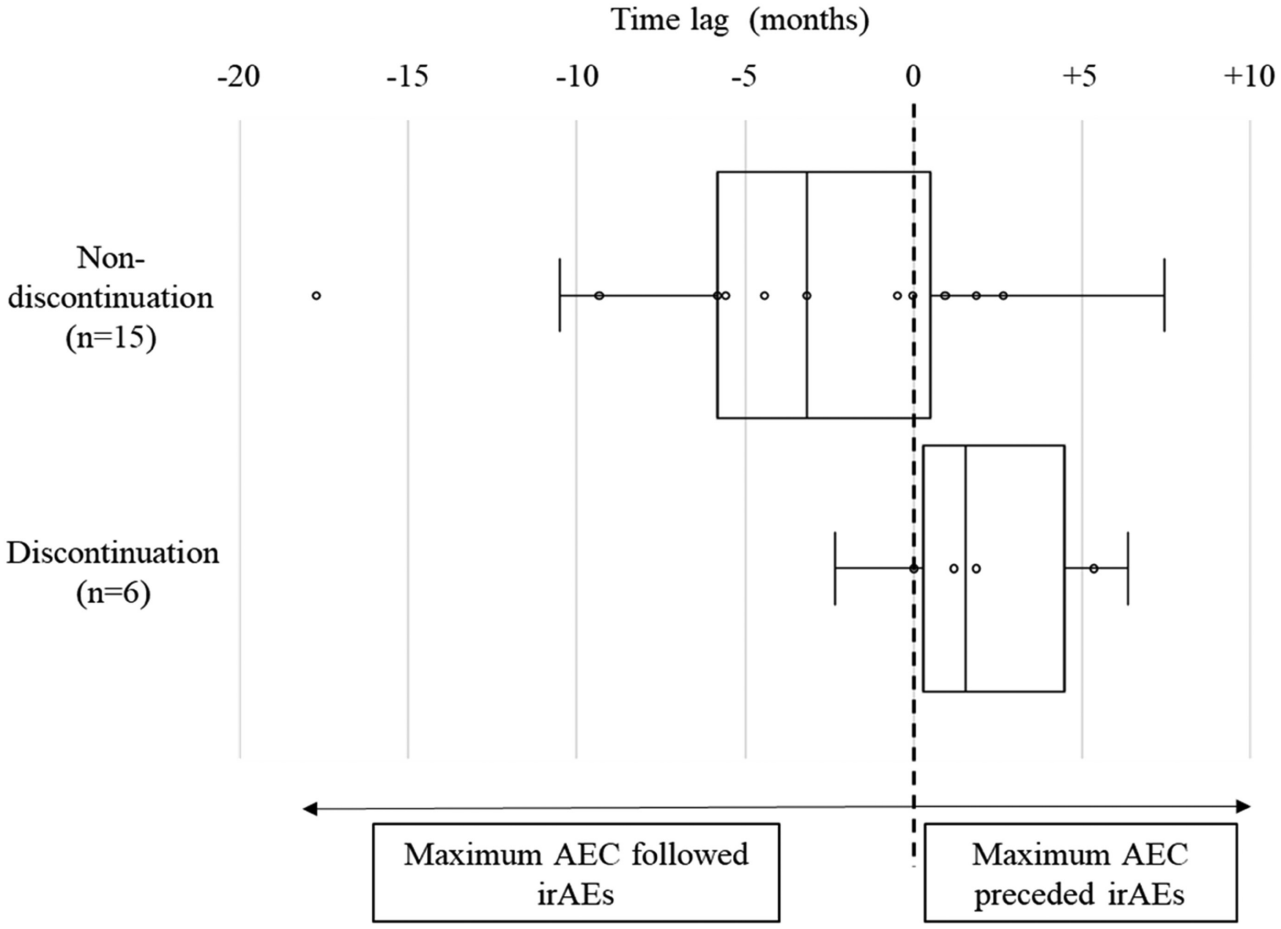
Time lag between the maximum absolute eosinophil count (AEC) and immune‐related adverse event (irAE) onset. The box‐and‐whisker plot shows the time lag between the maximum AEC and irAE onset. The upper column shows the time lag in the non‐discontinuation group. The median time lag was −3.2 (range, −17.7 to +7.5) months. The lower column shows the time lag in the discontinuation group. The median time lag was +1.5 (range, −2.3 to +6.4) months (*p* = 0.056). Time lag, time lag between the maximum AEC and irAE onset (months); middle vertical lines in columns, median time lag; left vertical lines in columns, first quartile; right vertical lines in columns, third quartile; left bars, minimum value; right bars, maximum value; circles, each data.

There were no significant differences between the irAE (+) and (−) groups in age, sex, recurrent/metastatic site, PS score, PD‐L1 expression level, prior radiotherapy, and number of prior chemotherapy lines (Table 4). However, the duration of nivolumab administration was significantly longer in the irAE (+) group than in the irAE (−) group (10.7 vs. 1.4 months, *p* < 0.001, Table 4). The maximum AEC of the irAE (+) group was significantly higher (548.8 vs. 182, *p* < 0.001) than that of the irAE (−) group, whereas the baseline AEC did not differ between the groups. In the irAE (+) group, the maximum AEC of the discontinuation group was significantly higher (729.3 vs. 368.6, *p* = 0.018) than that of the non‐discontinuation group (Table 4). The time to the maximum AEC was significantly longer in the irAE (+) group than in the irAE (−) group (5.8 vs. 0.9, *p* < 0.001) (Table 2). Figure 2 shows the time lag between the maximum AEC and irAE onset (irAE onset – maximum AEC) in the non‐discontinuation and discontinuation groups. The median time lag in the non‐discontinuation group was −3.2 (range, −17.7 to +7.5) months, whereas that in the discontinuation group was 1.0 (range, −0.4 to +6.3) months (*p* = 0.056, Table 4, Figure 2). Although not statistically significant (*p* = 0.056), the maximum AEC followed the irAEs in the non‐discontinuation group, whereas it preceded the irAEs in the discontinuation group.

**TABLE 4:** Predictive factors for irAEs and nivolumab discontinuation.

		irAE (−) (*n* = 41)	irAE (+) (*n* = 21)	*p*‐value	irAE (+) (*n* = 21)		*p*‐value
					Non‐discontinuation (*n* = 15)	Discontinuation (*n* = 6)	
Age	< 70	11	15	1	10	5	0.623†
	70 ≤	30	6		5	1	
Sex	Male	36	18	1	12	6	0.526†
	Female	5	3		3	0	
Recurrent/metastatic site	Locoregional	33	14	0.347	12	2	0.12†
	Distant metastasis alone	8	7		3	4	
Performance status	0,1	36	18	1	12	6	0.526†
	2 ≤	5	3		3	0	
PD‐L1 expression level	< 1% or not available	29	17	0.542	12	5	1†
	1% ≤	12	4		3	1	
Prior radiotherapy	Yes	29	13	0.57	9	4	1†
	No	12	8		6	2	
Number of chemotherapy lines	1,2	25	11	0.592	7	4	0.635†
	3	16	10		8	2	
Duration of nivolumab administration median (months, range)		1.4 (0.5–38)	10.7 (0.5–50.3)	**< 0.001**	12.4 (0.5–50.3)	5.9 (1.4–12.0)	0.095‡
Baseline AEC, median (range)		73.4 (0–1119.0)	117.7 (22.6–430.7)	0.24	94.6 (22.6–430.7)	158.5 (30.6–222.0)	0.34
Maximum AEC, median (range)		182 (0–1367.8)	548.8 (39.5–1218.0)	**< 0.001**	368.6 (39.5–869.4)	729.3 (541.1–1218.0)	**0.018**
Time to the maximum AEC, median (months, range)		0.9 (0–20.8)	5.8 (0.3–22.4)	**< 0.001**	6.8 (0.3–22.4)	4.3 (0.8–6.1)	0.275‡
Time to irAEs onset, median (months, range)		—	2.1 (0.6–14.7)	NA	1.4 (0.1–14.7)	6.1 (2.0–12.4)	0.129‡
Time lag between the maximum AEC and irAE onset (irAE onset – maximum AEC increasing), median (months, range)		—	0 (−17.7 to +7.5)	NA	−3.2 (−1.7 to +7.5)	1 (−0.4 to +6.3)	0.056‡

*Note*: Bold; statistically significant.

Abbreviations: AEC, absolute eosinophil count; ALC, absolute lymphocyte count; ANC, absolute neutrophil count; CRP, C‐related protein; irAEs, immune‐related adverse events; WBC, white blood cell.

^†^ Fisher's exact test.

^‡^ Mann–Whitney test.

### 3.6 Receiver operating characteristic curve of the maximum absolute eosinophil count (AEC) for predicting irAEs and nivolumab discontinuation

**FIGURE 3:**
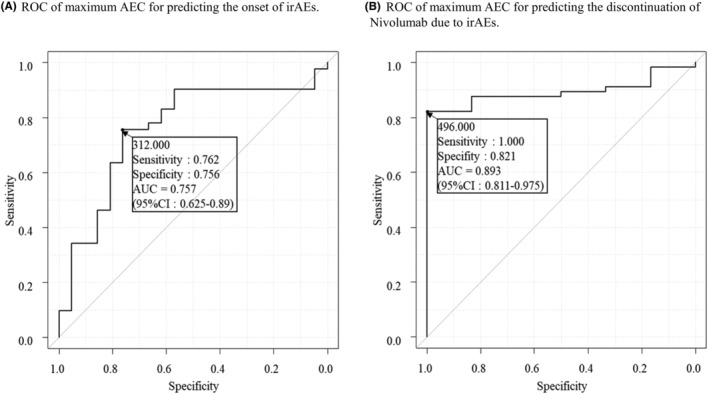
Predictive capability of absolute eosinophil count (AEC) for immune‐related adverse events (irAEs) and nivolumab discontinuation. (A) Receiver operating characteristic curve of AEC indicates irAE occurrence. The area under the curve was 0.757 (95% confidence interval [CI], 0.625–0.89), sensitivity was 0.762, and specificity was 0.756.(B) Receiver operating characteristic curve of AEC as an indicator of nivolumab discontinuation owing to irAEs. The area under the curve was 0.893 (95% CI, 0.811–0.975). The sensitivity and specificity were 1 and 0.821, respectively.

Figure 3A shows the ROC curve of the maximum AEC as a predictor of irAE occurrence. The area under the ROC curve was 0.757 (95% CI, 0.625–0.89). With a cutoff maximum AEC of 312, the sensitivity and specificity for predicting irAEs were 76.2% and 75.6%, respectively. Figure 3B shows the ROC curve of the maximum AEC for predicting nivolumab discontinuation owing to irAEs. The area under the ROC curve was 0.893 (95% CI, 0.811–0.975). With a cutoff maximum AEC of 496, the sensitivity and specificity for predicting discontinuation were 100% and 82.1%, respectively.

## 4 DISCUSSION

### 4.1 Survival outcomes, irAEs, and nivolumab discontinuation

In this study involving patients with R/M HNSCC who received nivolumab therapy, the survival durations were significantly longer in the irAE (+) group than in the irAE (−) group (Figure 1A,B). These results are consistent with those of a recent systematic review that demonstrated that irAE occurrence was associated with favorable treatment outcomes in various cancer types.^16^ Among patients with irAEs, those who discontinued nivolumab owing to irAEs (discontinuation group) achieved comparable survival to those who did not discontinue nivolumab (non‐discontinuation group) (Figure 1C,D). This could be partly explained by the high efficacy of salvage chemotherapy after ICI treatment.^17^, ^18^, ^19^ In addition, the durable effects of nivolumab, observed in the discontinuation group without sequential chemotherapy (Table 3), would also be responsible for the favorable survival outcomes. Similar durable response to nivolumab has been reported in non‐small cell lung carcinoma (NSCLC),^7^ metastatic renal cell carcinoma,^8^ and HNSCC.^9^ In patients with NSCLC, the binding of nivolumab to peripheral T cells previously treated with nivolumab is prolonged for more than 20 weeks after the last infusion,^20^ which may partly account for the durable effects of nivolumab.

### 4.2 Predictive factors for irAEs and nivolumab discontinuation

In the present study, we further investigated the predictive factors associated with irAEs and nivolumab discontinuation. In their meta‐analysis, Zhou et al. reported that high baseline AEC and a high neutrophil‐lymphocyte ratio/platelet‐lymphocyte ratio was significantly related to the risk of irAEs.^21^ Eosinophilia following the initiation of ICI treatment was also noted.^22^, ^23^ The current results showed that maximum AEC during nivolumab therapy, but not baseline AEC, was significantly higher in the irAE (+) group than in the irAE (−) group (548.8 vs. 182, Table 4), and had an area under the ROC curve of 0.757 (Figure 3A), demonstrating moderate accuracy in predicting irAE occurrence. In the irAE (+) group, the maximum AEC was significantly higher in the discontinuation group than in the non‐discontinuation group (729.3 vs. 368.6, Table 4). The maximum AEC had an area under the ROC curve of 0.893 (Figure 3B) for prediction of nivolumab discontinuation owing to irAEs, demonstrating high prediction accuracy. Maximum AEC was a significant predictor of irAEs and nivolumab discontinuation, which were associated with favorable survival outcomes. On analyzing data from the French pharmacovigilance database, Scanvion et al. found that 37 out of 1546 (2.4%) patients treated with ICIs had eosinophilia (≥1000).^23^ The median time to the AEC peak was 15 weeks, and 21 out of 37 (56.8%) patients manifested eosinophil‐related adverse events.^23^ While they observed patients with remarkable eosinophilia (≥1000) during ICI therapy, our results showed that patients who developed irAEs had mild eosinophilia (median, 548.8). Eosinophils infiltrate tumor tissues and regulate tumor progression either directly by interacting with tumor cells or indirectly by shaping the tumor microenvironment.^24^ Considering the latter, eosinophils release chemo‐attractants that induce the migration of tumor‐specific CD8+ T cells to the tumors, indirectly leading to tumor elimination.^25^ Certainly, migration of tumor‐specific CD8+ cells is also a favorable predictor for response to ICI therapy.^26^ These data support that not only baseline eosinophilia but also eosinophilia during ICI therapy may be an important factor for predicting irAEs and treatment outcome.

### 4.3 Chronological relationship between maximum AEC and irAEs

The chronological relationship between irAE onset and maximum AEC was different between the discontinuation and non‐discontinuation groups: The median time lag between the maximum AEC and irAE onset in the discontinuation group was +1 months, whereas it was −3.2 months in the non‐discontinuation group (Table 4, Figure 2). If irAEs occur at an early phase after the start of nivolumab (median, 1.4 months), the AEC would reach a peak later (median, 6.8 months), and irAEs would not result in nivolumab discontinuation (Table 4). In contrast, if the irAEs did not occur in the early phase after the start of nivolumab, a remarkable increase in AEC (median, 4.3 months) would precede the occurrence of severe irAEs, requiring nivolumab to be discontinued 1 month after the maximum AEC (Table 4). Consistent with the current results, it has been reported that eosinophilia precedes irAEs, such as hypopituitarism and adrenal deficiency, which develop more than 2 months after nivolumab administration.^27^, ^28^ All these findings suggest that an increase in AEC is involved in irAE occurrence and nivolumab discontinuation and that careful monitoring of the AEC can help predict the onset of severe irAEs, which is associated with favorable survival outcomes.

### 4.4 Limitations

This study has some limitations. First, this study was retrospective in nature and the sample size was small. Second, we could not exclude factors other than nivolumab administration that might be related to the eosinophil count (e.g., allergic rhinitis and bronchial asthma). Third, it is unclear whether eosinophilia and subsequent irAEs are characteristic to HNSCC. Future prospective studies are needed to determine how eosinophils affect PFS as well as irAE onset.

## 5 CONCLUSIONS

irAE occurrence was a favorable prognostic factor in patients with R/M HNSCC who receive nivolumab. Patients who discontinued nivolumab owing to severe irAEs had comparable treatment outcomes to those who continued nivolumab treatment. The AEC was significantly higher in patients with irAEs and in those who discontinued nivolumab owing to irAEs. Monitoring AEC during nivolumab therapy may be useful for predicting irAE occurrence, nivolumab discontinuation, and prognosis.

## AUTHOR CONTRIBUTIONS

**Yushi Ueki:** Conceptualization (equal); data curation (lead); investigation (lead); methodology (lead); writing – original draft (lead); writing – review and editing (equal). **Shusuke Ohshima:** Data curation (supporting); investigation (supporting); writing – review and editing (supporting). **Jo Omata:** Data curation (supporting); investigation (supporting); writing – review and editing (supporting). **Yusuke Yokoyama:** Data curation (supporting); investigation (supporting); writing – review and editing (supporting). **Takeshi Takahashi:** Data curation (supporting); investigation (supporting); writing – review and editing (supporting). **Ryusuke Shodo:** Data curation (supporting); investigation (supporting); writing – review and editing (supporting). **Keisuke Yamazaki:** Data curation (supporting); investigation (supporting); writing – review and editing (supporting). **Arata Horii:** Conceptualization (equal); methodology (supporting); supervision (lead); writing – original draft (supporting); writing – review and editing (equal).

## FUNDING INFORMATION

None declared.

## CONFLICT OF INTEREST STATEMENT

The authors declare no conflicts of interest.

## ETHICAL APPROVAL STATEMENT

This study was approved by the Institutional Review Board of Niigata University Hospital (No. 2019–0172). This study was conducted in accordance with the principles of the Declaration of Helsinki. The patients provided their written informed consent to participate in this study.

## Data Availability

The original contributions presented in the study are included in the article/supplementary material; further inquiries can be directed to the corresponding author.
